# Distal femoral shortening osteotomy for managing irreducible hips during total hip replacement in four dogs with severe luxoid hips

**DOI:** 10.1111/vsu.14257

**Published:** 2025-03-31

**Authors:** YoungJin Jeon, Jaemin Jeong, Aldo Vezzoni, Haebeom Lee

**Affiliations:** ^1^ Department of Veterinary Surgery, College of Veterinary Medicine Chungnam National University Daejeon Republic of Korea; ^2^ Clinica Veterinaria Vezzoni srl Cremona Italy

## Abstract

**Objectives:**

To describe the surgical technique and clinical outcomes of distal femoral shortening osteotomy (DFSO) to facilitate prosthesis reduction in dogs with irreducible luxoid hips undergoing total hip replacement (THR).

**Animals:**

Four client‐owned dogs with luxoid hips.

**Study design:**

Short case series.

**Methods:**

Standard THR was performed to alleviate pain and restore limb function. After confirming the prostheses were non‐reducible, DFSO was performed as a novel tension‐relieving technique at a level that allowed internal fixation. The shortening length was determined by the intraoperative tension required to reduce and maintain the prosthesis. Data from medical records were collected, including signalment, clinical signs, implant used, shortening length, and outcomes.

**Results:**

Following DFSO, prosthesis reduction was successful in all dogs. The median femoral shortening length ratio was 13.8% (range, 10.7%–15.3%). One intraoperative complication involved a fissure of the greater trochanter, which occurred during trial reduction before DFSO. Median duration of follow‐up was 21 months (range, 3–34 months). Two dogs showed good to excellent limb function. Bone union was consistently achieved in all DFSO procedures. Two postoperative complications were observed: one case of prosthesis luxation and one case of aseptic stem loosening. The owner declined revision surgery for luxation, and explantation was performed for the stem loosening.

**Conclusion:**

DFSO could effectively manage irreducible prostheses in dogs with luxoid hips when conventional methods fail. However, careful case selection and meticulous surgical planning were essential to avoid complications.

## 1 INTRODUCTION

Luxoid hip represents a severe form of hip dysplasia characterized by atraumatic hip luxation.^1^ This condition can lead to significant clinical consequences, such as joint ankylosis, pain and severe lameness. Total hip replacement (THR) is considered the optimal surgical treatment for dogs with luxoid hips when clinical signs are refractory to conservative treatment.^2^

However, THR presents several perioperative challenges in these conditions due to anatomical deformation in the hip joint and increased soft tissue tension. In particular, fibrotic tissue formation around the joint and shortening of periarticular muscles significantly increase the risk of femoral fracture during prosthesis reduction or may render prosthesis reduction impossible.^3^, ^4^ Although a previous study has reported complications related to femoral fractures during prosthesis reduction in luxoid hips,^3^ the issue of irreducible prostheses and the surgical techniques to address these challenges remains underexplored in the veterinary literature.

wIn human medicine, a similar condition called high‐riding hips has been documented. In such cases, irreducible prostheses and neurovascular damage are notable concerns during joint replacement.^5^ Consequently, numerous studies have focused on classifying and developing surgical techniques to manage these cases.^5^, ^6^, ^7^, ^8^ Among the methods, subtrochanteric shortening osteotomy and distal femoral shortening osteotomy (DFSO) have been used in people to alleviate soft tissue tension and facilitate prosthesis reduction.^5^, ^8^ However, to our knowledge, there are no reports on the use of DFSO in veterinary medicine, particularly in addressing luxoid hips in dogs. DFSO was chosen because it was considered a more appropriate surgical method for dogs, as it could provide strong fixation due to the preservation of the proximal femoral anatomical structure.^8^ In addition, stem fixation is performed in the proximal part of the femur, and therefore we considered it appropriate to perform the osteotomy in the distal femur and not in the proximal femur.

This case series employed distal femoral shortening osteotomy to address irreducible prostheses in four dogs with luxoid hips. Our objective was to document the surgical technique, explore potential surgical indications, and report the outcomes of DFSO in dogs undergoing cementless THR.

## 2 MATERIALS AND METHODS

### 2.1 Cases and clinical evaluation

Four dogs were presented for surgical treatment of luxoid hips. Three dogs showed grade III/IV and one dog (dog 3) was evaluated as grade II/IV pelvic limb lameness.^9^ Physical examination revealed pain during passive range of motion (PROM) of hip joint, particularly during extension and abduction. Even under heavy sedation, the craniodorsally dislocated femoral head could not be reduced by direct distal‐medial pressure on the greater trochanter. All dogs were diagnosed with luxoid hips, characterized by hip dysplasia with complete dislocation of the femoral head beyond the margins of the ilium and ischium at the acetabular level on lateral pelvic radiography (Table 1). Three cases used the Zurich Cementeless Hip Replacement system (Kyon AG, Zurich, Switzerland) and one case (dog 4) used the BFX system (BioMedtrix Universal Hip System, BioMedtrix, Boonton, New Jersey).

**TABLE 1:** Preoperative clinical information.

Dog	Breed	Age (months)	Sex	BW (kg)	Body condition score	Luxation duration (months)	FHD (%)	PFV angle	Diagnosis	Other orthopedic conditions
1	Belgian sheepdog	13	Castrated male	24	5/9	10	153.1	8.1	Bi. luxoid hips CHD	PFV deformity
2	Alaskan Malamute	49	Castrated male	37.8	5.5/9	At least 1 yr	110	1.2	Bi. luxoid hips CHD, OA	None
3	Golden Retriever	24	Spayed female	26.8	6/9	At least 1 yr	45.1	3.9	Uni. luxoid hip with CHD, OA	None
4	Golden Retriever	61	Castrated male	28.5	5/9	At least 3 yr	101.1	4.1	Uni. luxoid hip CHD, OA	None

Abbreviations: Bi., bilateral; BW, bodyweight; CHD, chronic hip dysplasia; FHD, height of the femoral head displacement; OA, osteoarthritis; PFVA, proximal femoral valgus; Uni., unilateral.

### 2.2 Radiographic evaluation

**FIGURE 1:**
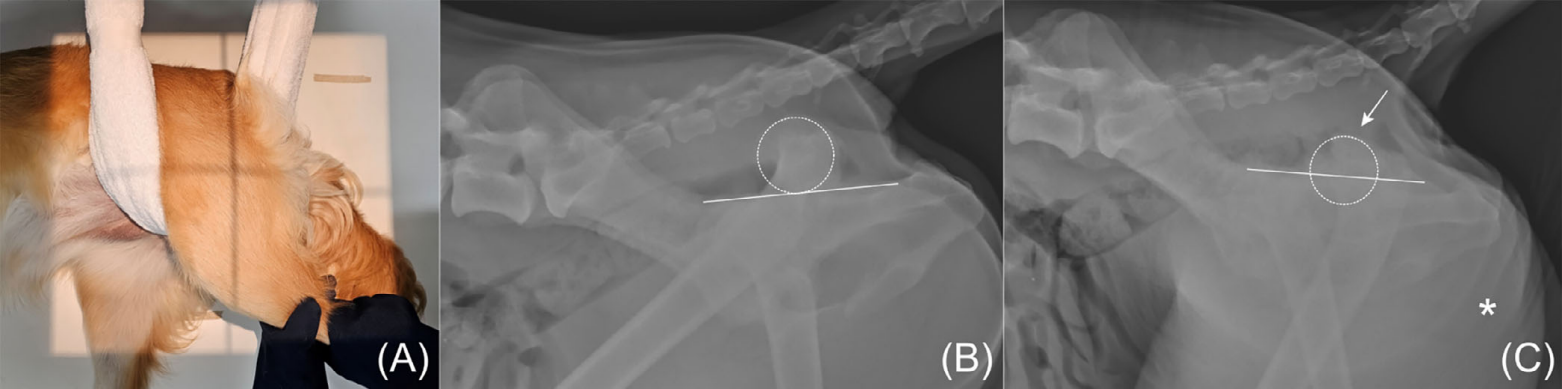
Preoperative images to evaluate the amount of dorsal displacement. (A) Modified lateral pelvic radiograph was taken by distracting the limb distally. (B) Conventional lateral pelvic view of dog 4, showing the femoral head dislocated dorsally (dashed circle) with a dislocated femoral head index (FHD) of 101.1%. (C) Modified lateral pelvic view of the same dog. Despite the traction, the femoral head could not be tracked distally. FHD was measured as 61.4%. The white arrow marks the externally rotated femur, and the asterisk indicates a skin fold caused by the assistant's towel.

All dogs were deeply sedated for imaging. Four standard radiographs for THR were taken.^2^ Proximal femoral morphology was assessed the angle between the proximal femoral axis and the femoral anatomic axis as variations in this angle may influence implant behavior and load distribution.^3^ A proximal femoral angular deformity was categorized if the angle exceeding 5°.^3^ Femur length was measured from the most proximal extent of the greater trochanter to the distal border of trochlea on the craniocaudal projection. The proximodistal height of the femoral head displaced (FHD) was calculated as the percentage of the proximodistal distance of the femoral head, measured beyond the margin of the ilium and ischium at the acetabulum level, relative to the femoral head diameter on a lateral pelvic radiograph with superimposed ilia.^3^ FHD was measured to assess the severity of dislocation (Figure 1B). In addition, a modified pelvic lateral view was devised to evaluate the magnitude of soft tissue tension and performed in one dog (Figure 1A,C). Modifications from the conventional lateral view included external rotation, slight adduction of the femur, and distal traction of the femur, mimicking the manipulation used to reduce the luxated hip. FHD was measured on the modified pelvic lateral view and compared with the FHD of the conventional view. All measurements on radiographs were performed on the surgical planning program (vPOP PRO, Vet SOS Education Limited, Shrewsbury, UK).

### 2.3 Preoperative radiographic findings

Radiographs showed that the femoral head was dislocated from the true acetabulum in all four dogs. The median FHD was 105.5% (range, 45.1%–153.1%). In dog 4, using the modified pelvic lateral radiograph, the center of the femoral head could not move under the dorsal margin between the ilium and ischium. The FHD was measured at 61.4% on this view, compared to 101.1% on the conventional view. A proximal femoral valgus of 8.1° was measured in dog 1. This information is listed in Table 1.

### 2.4 Surgical procedure

The standard THR technique was performed. Despite employing conventional tension‐relieving techniques, including rectus femoris and pectineus release, and lowering the neck osteotomy line, prosthesis reduction remained impossible. Consequently, DFSO was performed.

**FIGURE 2:**
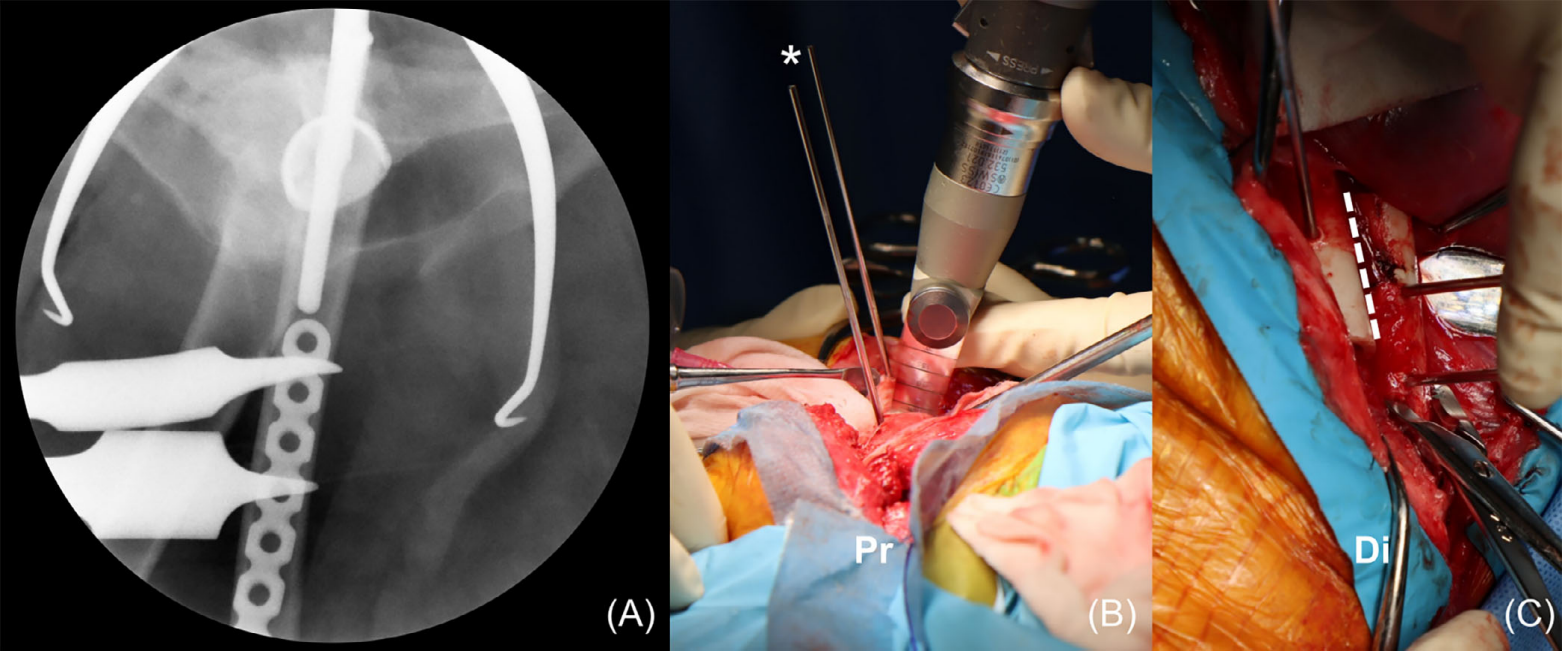
Intraoperative images of distal femoral shortening osteotomy. (A) Fluoroscopy was used to plan the first osteotomy level and plate position. (B) Jig pins (asterisk) placed before the osteotomy to facilitate reduction, compression of the osteotomy line, and alignment of the femur. (C) The second osteotomy line was determined after prosthesis reduction to maintain joint stability. The dashed line highlights the overlapping of femoral segments. Pr, proximal; Di, distal.

The first osteotomy level was determined preoperatively using a computer‐assisted surgical planning program and intraoperatively confirmed via fluoroscopy to ensure sufficient bone stock for screw placement between the distal femoral stem tip and the osteotomy line (Figure 2A). Before performing the first osteotomy, two alignment pins for the jig were positioned on the lateral surface of the femur, one proximal and one distal to the osteotomy line, both placed parallel to each other (Figure 2B). After the first osteotomy, the proximal femoral segment was intentionally displaced laterally to overlap with the distal femoral segment, enabling prosthesis reduction (Figure 2C). The distal segment was manually advanced distally while evaluating the stability of the prosthetic reduction and the muscular tension, ensuring the femur remained perpendicular to the pelvis and the stifle could be flexed. Subsequently, a second osteotomy was performed to remove the overlapping bone between the proximal and distal segments. The osteotomized femur was then stabilized using a locking plate‐screw system (Table 2), aligned with the jig placement. Orthogonal plating, in which a larger main plate (3.5 mm locking plate) was placed laterally and a smaller plate (2.7 mm locking plate) cranially, was applied in all cases. The surgical site was irrigated with sterile saline, and a culture swab was taken. Standard closure procedures were performed.

**TABLE 2:** Postoperative clinical information.

Dog	THR system	Prosthesis	ALO (°)	SL/SLR (mm/%)	Cup retroversion angle (°)	Internal fixation	Intra‐/postoperative complication	Last follow‐up (months)	Outcome
1	Zurich	26 mm cup, S stem, XS neck, 19 mm head	40	30.9/14.3	15.1	3.5mm^a^ 6‐hole poly‐axial LP 2.7 mm^a^ 6‐hole poly‐axial LP 2.7 mm long poly‐axial LP (from GT to distal femur, laterally)	Fissure^b^ on GT/aseptic stem loosening	18	Explantation
2	Zurich	26.5 mm revision cup, M stem, M neck, 19 mm head	49	21.8/10.7	24.1	3.5 mm 8‐hole poly‐axial LP 2.7 mm 6‐hole poly‐axial LP	None/none	34	Satisfactory
3	Zurich	26.5 mm revision cup, S stem, S neck, 19 mm head	52	25.3/13.3	18.3	3.5 mm 6‐hole poly‐axial LP 2.7 mm 4‐hole poly‐axial LP	None/none	24	Satisfactory
4	BioMedtrix BFX	24 mm cup, No.7 stem, 17 mm (+0) head	45	29.6/15.3	16.7	3.5 mm 12‐hole LP (from GT to distal femur, laterally) 2.7 mm 6‐hole poly‐axial LP	None/prosthetic luxation	3	Declined revision surgery

Abbreviations: ALO, angle of lateral opening; GT, greater trochanter; LP, locking plate; SL/SLR, shortening length/ratio; THR, total hip replacement.

^a^ 3.5 and 2.7 mm bone plates were placed on the lateral and cranial surfaces of the femur, respectively.

^b^ During trial reduction before osteotomy.

### 2.5 Surgical findings

The prosthesis was successfully reduced following DFSO in all cases. The implant sizes for each patient are listed in Table 2. In dogs 2 and 3 with severe dysplastic wear of the dorsal acetabular rim present, a revision‐type cup (KYON) was used. In dog 1, a fracture occurred at the greater trochanter during the trial reduction before DFSO. A poly‐axial locking plate and a double‐loop cerclage wire were applied to stabilize the greater trochanter. Based on the surgeon's decision, in dog 4, a long lateral plate was applied from the greater trochanter to the distal femoral segment. No intraoperative complications were observed in the other dogs.

### 2.6 Postoperative management

Orthogonal radiographs were taken immediately after surgery. Meloxicam (0.1 mg/kg subcutaneously every 24 h) and gabapentin (15 mg/kg orally every 12 h) were administered for pain control during hospitalization. The patients were able to stand and ambulate with the support of a sling the day after surgery. All patients were discharged 7 days post‐surgery, and cage rest was prescribed for 6–8 weeks. Afterwards, restricted activity and leash‐walk with a sling were advised for 2 months postoperatively. A gradual increase in activity was recommended following follow‐up evaluations. Follow‐ups were conducted at 39, 67, 103, 177, 288, and 355 days for dog 1; 23, 66, 106, 132, 695, and 1021 days for dog 2; 25, 67, 88, 137, 223, 301, and 710 days for dog 3, all postoperatively and at the hospital; and at 42 and 93 days for dog 4, postoperatively at a local hospital. At each follow‐up, routine physical and orthopedic examinations, combined with gross gait analysis and orthogonal radiographs of the hip, were performed.

## 3 RESULTS

### 3.1 Clinical outcomes and postoperative complications

No complications occurred during hospitalization. Callus formation or resolution of the osteotomy line was confirmed in all cases by 3 months post‐surgery. No evidence of neurovascular damage was observed during the follow‐ups. At the last follow‐up, lameness score improved to grade I/IV in two dogs (dog 3 and 4) and grade II/IV in dog 2. The owners reported satisfaction with the limb function. Pain was absent during PROM of the prosthetic hip joint in all dogs except dog 1. However, pain persisted on the contralateral side.

**FIGURE 3:**
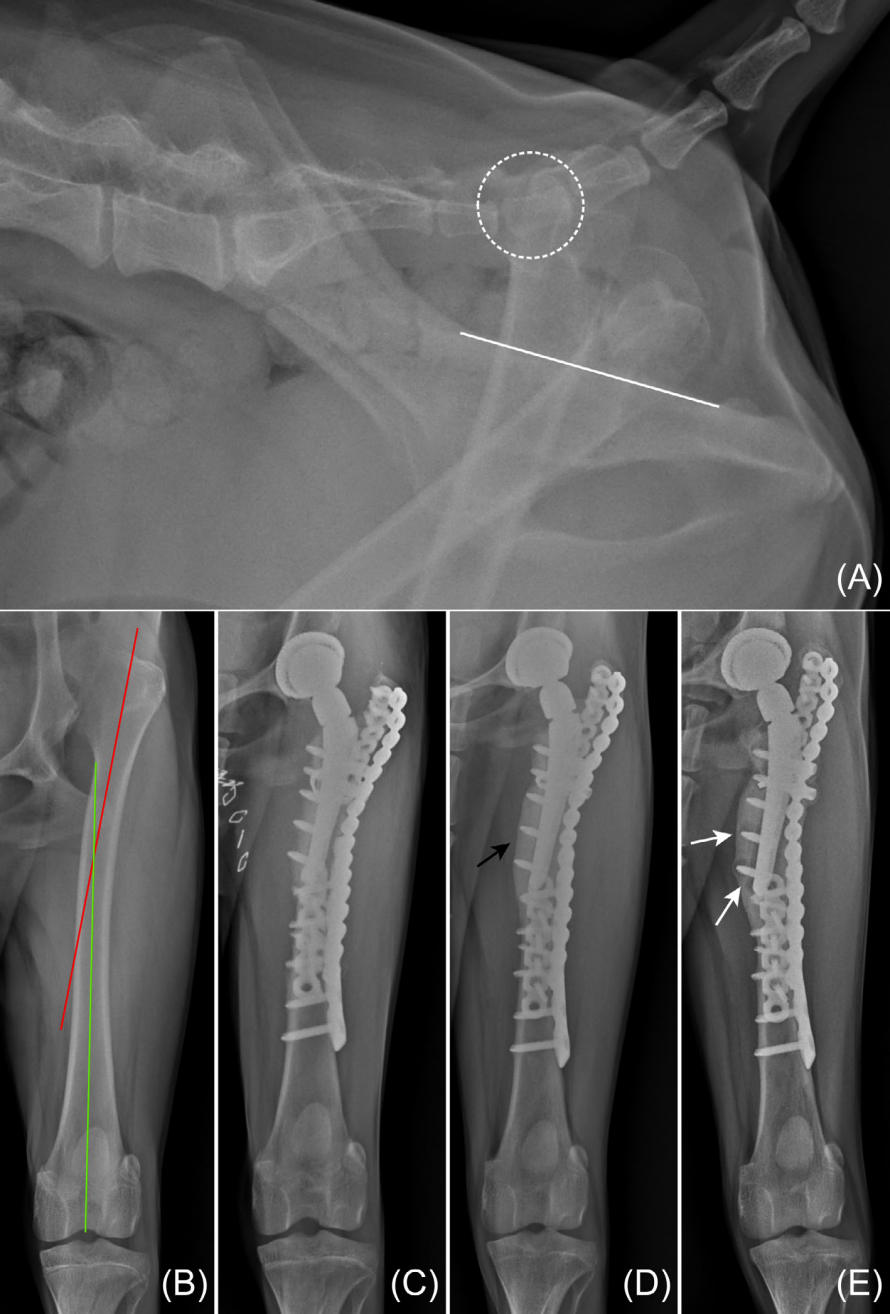
Perioperative radiographs of the femur in dog 1. (A) Preoperative lateral pelvic view shows a dislocated femoral head index of 153.1%. (B) The proximal femoral valgus deformity is evident, measured at 8.1°. (C) The immediate postoperative craniodorsal radiograph reveals varus stem position. (D) At 103 days post‐surgery follow‐up, progression of varus stem position and thickening of the proximomedial cortex (black arrow) were confirmed. (E) A radiograph taken one‐year post‐surgery reveals radiolucent cavities (white arrows) around the screws and stem.

Dog 1 showed a weightbearing lameness 1 month after surgery. Craniocaudal femur radiographs showed worsening of the stem valgus angulation, increasing from 10.1° immediately post‐surgery to 12.4° at 1 year postoperatively, along with thickening of the proximo‐medial cortex of the femur (Figure 3B–E). Pain was elicited from full extension of hip joint. Loosening of the femoral component was confirmed at the one‐year follow‐up, followed by explantation of the prostheses. During the explantation, bacterial culture samples were obtained from the periprosthetic tissue and analyzed using sonication techniques. The results were negative, suggestive for aseptic stem loosening. Prosthetic luxation occurred in dog 4 6 weeks after surgery during a radiography examination at a local animal hospital. After a closed reduction, the dog showed good limb function 3 months post‐surgery. However, reluxation occurred due to the same cause, and the owner declined further surgical intervention.

### 3.2 Postoperative radiographic evaluation

**FIGURE 4:**
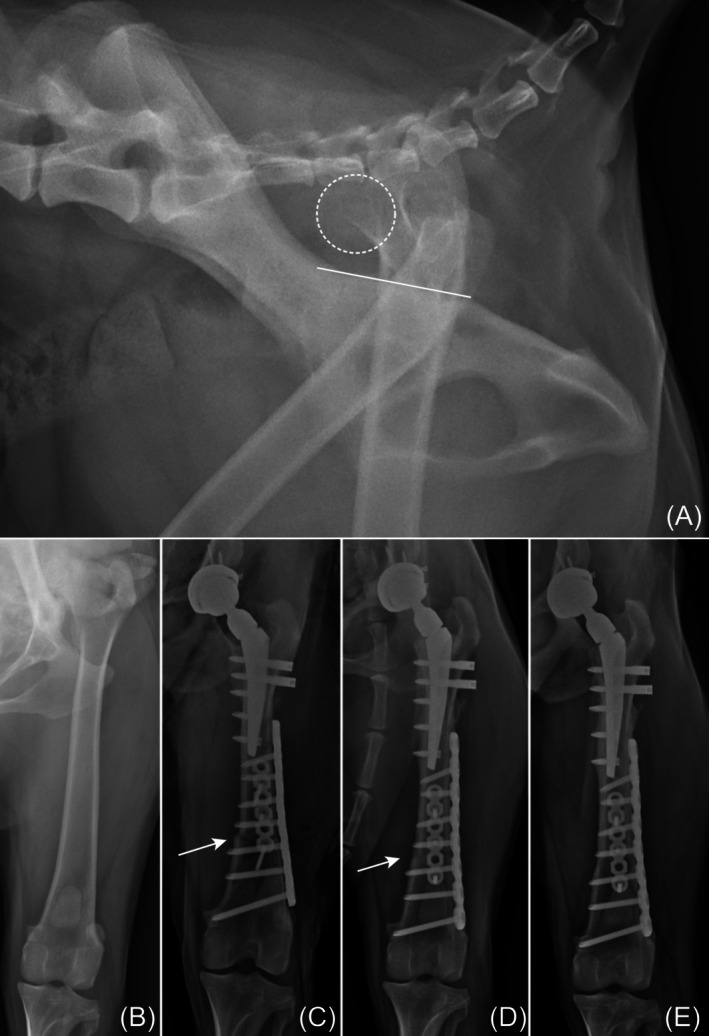
Perioperative radiographs of the femur in dog 2. (A) Preoperative lateral pelvic view shows a dislocated femoral head index of 110%. (B) Preoperative craniodorsal view shows no evident deformities. (C) Immediate postoperative radiograph shows the distal femoral shortening osteotomy (white arrow). (D) By 106 days post‐surgery, the osteotomy line is no longer visible. (E) Radiograph at 695 days post‐surgery shows no signs of implant failure.

**FIGURE 5:**
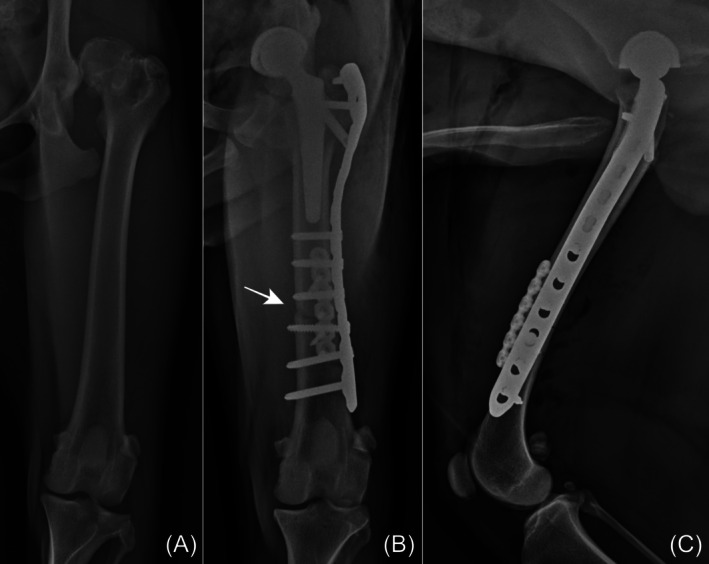
Perioperative radiographs of the femur in dog 4. (A) Preoperative craniodorsal view shows dislocation of the femoral head. (B, C) Immediate postoperative radiograph shows the osteotomy line (white arrow) fixed with a long lateral locking plate and a short cranial plate, along with the BFX system.

There were no significant findings on postoperative radiographs for all dogs except dog 1 (Figures 3, 4, 5). The stem was implanted in a valgus position, measured at an angle 10.1°. The other dogs had a median stem varus angulation of 1.75° (range, 1.1–2.7°). The median angle of the cup lateral opening and the femur shortening ratio were 47° (range, 40–52°) and 13.8% (range, 10.7%–15.3%), respectively. The median cup version angle was 17.5° (range, 15.1–24.1°). Individual measurements for each dog are listed in Table 2.

## 4 DISCUSSION

In this case series, we describe the surgical techniques, physical assessments, and radiographic evaluations of dogs with severe luxoid hips undergoing DFSO as part of cementless THR to manage irreducible prostheses. Although two dogs experienced postoperative complications—one with aseptic stem loosening and another with prosthetic luxation—DFSO appears to be a viable technique for managing irreducible prostheses during THR in cases of severe femoral head dislocation, even when conventional tension‐relieving methods are insufficient.

Several studies have addressed luxated hips treated with THR in dogs. Two studies reported that no modified technique was necessary for reducing the prostheses,^3^, ^10^ indicating that significant tension and leverage to fatigue the soft tissues were sufficient for reduction.^10^ However, in some cases, femoral fractures occurred during prosthesis reduction.^3^, ^10^ In contrast to these studies, our cases could not be reduced despite the conventional tension‐relieving techniques. In our cases, the mean FHD on conventional radiographs was 102.32% ± 44.39%, nearly double the value reported in a previous study.^3^ In our cases, the severity of femoral head dislocation and increased periarticular soft tissue tension may have been greater than in previously reported cases.

Given the need for an objective evaluation of periarticular tension and reducibility, modified lateral radiographs involving distal traction of the femur under deep sedation were employed in this study. Although only one case was evaluated, the FHD assessed from the modified lateral pelvic radiograph in dog 3 was 61.4%, whereas the FHD on conventional lateral pelvic radiography was 101.1%. The FHD values on these modified radiographs, which reflect the magnitude of tension and dislocation, could serve as criteria for DFSO. Further studies with larger sample sizes are required to establish quantitative criteria for DFSO. Until such criteria are defined, intraoperative confirmation of irreducible prostheses remains the primary indication for DFSO.

In human medicine, studies on osteotomy techniques, such as subtrochanteric osteotomy and DFSO, have addressed irreducible prostheses in high‐riding hips.^5^, ^6^, ^7^, ^8^ However, several complications are associated with subtrochanteric osteotomy, including femoral fractures, nerve injuries, delayed union, nonunion, and loosening of the femoral implant.^5^, ^6^, ^7^, ^8^ These complications may arise because the osteotomy level in subtrochanteric osteotomy is located at the midshaft area of the proximal femur, which is crucial for resisting micromotion under high torsional loads. Its disruption may compromise structural integrity, potentially leading to instability and complications.^8^ Koulouvaris et al. explained that DFSO preserves proximal femoral anatomy.^8^ This theoretical rationale could apply to using DFSO in dogs instead of subtrochanteric osteotomy.

Stress riser fractures are another potential complication associated with femoral osteotomy. The proximity of the stem and plate, particularly in cases where the plate is positioned immediately distal to the stem or overlaps insufficiently (Figure 4C–E), may exacerbate stress concentration and predispose the femur to stress riser fractures. Biomechanical research in human medicine recommends placing the plate either overlapping the stem by at least two shaft diameters with a proximal screw or at least 6–10 mm distal to the stem tip to reduce the risk of such fractures.^11^, ^12^, ^13^ Although no complications related to bone healing or interprosthetic fractures were observed in our cases, applying these principles to veterinary applications may help mitigate the risk of stress riser fractures. For instance, using a long lateral locking plate in DFSO procedures could reduce such risks, particularly in young dogs with softer bone or when utilizing the Kyon system, as gliding holes could act as stress risers. Further studies are warranted to assess the effectiveness of these approaches in veterinary patients.

The theoretical rationale of DFSO is that the procedure releases muscles originating from the pelvis and inserting around the stifle, such as the adductor, rectus femoris, and semitendinosus, which resist distal femoral translation and prosthetic reduction. In contrast, muscles originating from the pelvis and inserting into the proximal femur, such as the deep and middle gluteal muscles, external obturator, and gemelli, are not affected by DFSO and may theoretically oppose hip reduction due to their natural tension. However, in our cases, these proximal muscles did not appear to interfere with prosthetic reduction after femur shortening. Furthermore, DFSO may reduce the risk of nerve palsy by avoiding excessive soft tissue tension. While there is limited information regarding nerve damage due to soft tissue tension after THR for luxoid hips in dogs, limb lengthening exceeding 4 cm is known to be a risk factor for nerve damage in human medicine.^5^ Despite dorsal femoral displacement distances from the true acetabulum exceeding 4 cm in two of our dogs (dogs 1 and 2), no complications associated with nerve damage were observed. Further studies are necessary to evaluate the potential association between limb lengthening and nerve damage and determine the optimal shortening length for performing DFSO.

A recent study confirms that LH is a high‐risk factor for major complications.^3^ These complications likely result from multifactorial issues, including excessive tension, proximal femoral deformity, and acetabular cup orientation. In our cases, aseptic loosening and prosthetic luxation each occurred. The subperiosteal formation of new bone observed in dog 1 may have resulted from micromovement due to implant loosening associated with the intraoperative trochanteric fracture.^14^ Increased stem valgus angulation and lower radiopacity around screws also indicated loosening. Risk factors for stem loosening include improper femoral stem size, and excessive polyethene wear, which is associated with component impingement.^15^ There is a lack of literature correlating stem malposition with loosening in the Zurich THR system, but human studies describe such a relationship. Proximal femoral deformity can compromise stem position, complicate osteointegration, and increase the risk of fractures.^16^ In our case, the proximal femoral valgus deformity was not initially considered during surgical planning, which likely contributed to the malposition of the stem. This represents a missed opportunity, as addressing the deformity through corrective osteotomy during the procedure could have improved implant positioning, stability, and overall surgical outcomes. Therefore, corrective osteotomy or a custom stem should be considered in cases of proximal femoral deformity.^17^ Moving forward, thorough preoperative assessment of femoral conformation should be emphasized to optimize surgical planning for similar cases.

This report had several limitations. The small sample size and retrospective nature of the data are significant limitations. While capturing the modified pelvic lateral view, external rotation was applied. However, unlike traumatic luxation, the luxoid hip is unlikely to be hooked on the dorsal iliac spine, and the dislocated femoral head is usually well lateral to the acetabulum. Therefore, external rotation may have been unnecessary and could even interfere with the assessment of periarticular tension. Additionally, there are no established indications or osteotomy levels for DFSO, with the second osteotomy line determined based on the surgeon's judgment and experience. A particular concern is the role of postoperative soft tissue tension, which is crucial for maintaining the prosthetic hip joint. Soft tissue tension is directly related to the shortening length and should be studied further to reduce the risk of prosthetic luxation. In dog 4, the luxation was suspected to result from reduced soft tissue tension because of excessive femoral shortening, highlighting the importance of this factor in postoperative outcomes. Further research is required to establish quantitative criteria for osteotomy planning in DFSO. While CT‐based three dimensional (3D) imaging and preoperative planning tools have been proposed for human total hip arthroplasty, particularly in the context of limb length discrepancy and related shortening osteotomy, their application to evaluate periarticular tension and its relationship to prosthetic reducibility has not yet been explored.^18^ Additionally, CT imaging would be particularly beneficial for luxoid dogs with femoral deformities like dog 1, allowing precise assessment of the deformity and planning of corrective osteotomy. Future studies should investigate the feasibility of using advanced imaging technologies to assess periarticular tension and optimize osteotomy planning in veterinary patients.

We describe a novel technique for reducing THR prostheses in dogs with luxoid hips. DFSO, paired with THR, could be a viable option to facilitate prosthesis reduction in dogs with severe luxoid hips. Further studies are needed to standardize the technique and reduce complications.

## AUTHOR CONTRIBUTIONS

Jeon YJ, DVM, MS: Design, acquisition and interpretation of data, and drafting of the work. Jeong JM, DVM, MS, PhD: Concept and design of work, acquisition and interpretation of data, drafting of the work, and critical review of the work. Vezzoni A, DVM, SCAMPA, DipECVS: Concept and design of work, interpretation of data, critical review of the work. Lee HB, DVM, MS, PhD, DAiCVS: Concept and design of work, acquisition and interpretation of data, perform the surgery, and critical review of the work. All authors provided a critical review of the manuscript and endorsed the final version. All authors are aware of their respective contributions and have confidence in the integrity of all contributions.

## FUNDING INFORMATION

The Korea Institute of Planning and Evaluation for Technology in Food, Agriculture and Forestry, and the Ministry of Agriculture, Food and Rural Affairs, Grant/Award Number: MAFRA 322090.

## CONFLICT OF INTEREST STATEMENT

The authors declare no conflicts of interest related to this report.
